# A Novel Role for Polycystin-2 (Pkd2) in *P. tetraurelia* as a Probable Mg^2+^ Channel Necessary for Mg^2+^-Induced Behavior

**DOI:** 10.3390/genes10060455

**Published:** 2019-06-14

**Authors:** Megan S. Valentine, Junji Yano, Judith Van Houten

**Affiliations:** 1 State University of New York at Plattsburgh, 101 Broad Street, Plattsburgh, NY 12901, USA; 2 Department of Biology, University of Vermont, 120 Marsh Life Science, 109 Carrigan Drive, Burlington, VT 05405, USA; junji.yano@uvm.edu (J.Y.); Judith.Vanhouten@uvm.edu (J.V.H.)

**Keywords:** Polycystin-2, channelopathy, cilia, cell body membrane, magnesium channel, electrophysiology, behavior, over-expression, trafficking

## Abstract

A human ciliopathy gene codes for Polycystin-2 (Pkd2), a non-selective cation channel. Here, the Pkd2 channel was explored in the ciliate *Paramecium tetraurelia* using combinations of RNA interference, over-expression, and epitope-tagging, in a search for function and novel interacting partners. Upon depletion of Pkd2, cells exhibited a phenotype similar to *eccentric* (XntA1), a *Paramecium* mutant lacking the inward Ca^2+^-dependent Mg^2+^ conductance. Further investigation showed both Pkd2 and XntA localize to the cilia and cell membrane, but do not require one another for trafficking. The XntA-myc protein co-immunoprecipitates Pkd2-FLAG, but not vice versa, suggesting two populations of Pkd2-FLAG, one of which interacts with XntA. Electrophysiology data showed that depletion and over-expression of Pkd2 led to smaller and larger depolarizations in Mg^2+^ solutions, respectively. Over-expression of Pkd2-FLAG in the XntA1 mutant caused slower swimming, supporting an increase in Mg^2+^ permeability, in agreement with the electrophysiology data. We propose that Pkd2 in *P. tetraurelia* collaborates with XntA for Mg^2+^-induced behavior. Our data suggest Pkd2 is sufficient and necessary for Mg^2+^ conductance and membrane permeability to Mg^2+^, and that Pkd2 is potentially a Mg^2+^-permeable channel.

## 1. Introduction

Polycystin-2 (PC2, Pkd2, or TRPP1) is the protein known to be a product of a channelopathy gene, whose mutations are responsible for ~15% of the cases of autosomal dominant polycystic kidney disease (ADPKD), and is thought to be an ion channel homologous to members of the P-type subfamily of transient receptor potential (TRP) proteins [1,2]. There are 28 TRP proteins grouped into six subfamilies: TRPM (melastatin), TRPC (canonical), TRPV (vanilloid), TRPA (ankyrin), TRPML (mucolipin), and TRPP (polycystin). The members of the TRP family share a structural homology of six predicted transmembrane-spanning regions with a pore region between S5 and S6 along with varying lengths of their intracellular N- and C- termini, reviewed in [3]. Three of the subfamilies, TRPC, TRPV, and TRPM, are similar in their predicted structure and in their sequence homology while the remaining subfamilies have more divergent sequences and predicted structures, including the TRPP subfamily containing the polycystin proteins. 

A second TRPP protein, polycystin-1 (Pkd1) differs from Pkd2 in structure. While the Pkd2 protein has the traditional TRP structure of six transmembrane spanning regions and a pore region between S5 and S6, the Pkd1 protein [4,5,6,7] with a molecular mass of over 450 kD and 11 transmembrane spanning regions is not considered a true TRP channel [4]. The Pkd1 and Pkd2 proteins have been investigated for their interactions with one another, which are important for mechanosensation or trafficking [8,9,10,11,12,13]. A recent analysis of mutations in the *PKD1* or *PKD2* genes leads to ~87% and 13%, respectively, of the cases of autosomal dominant polycystic kidney disease (ADPKD) [14]. ADPKD is one of the leading causes of adult-onset renal failure in the United States, affecting approximately one out of every 800 live births [15,16].

The Pkd2/PC2 protein is a non-selective cation channel in mammalian cells. Recordings from primary cilia of murine epithelial cells from the renal inner medullary collecting duct show native PC2 is permeable to K^+^ and Ca^2+^ more so than to Na^+^ [17]. Human Pkd2 protein expressed in reconstituted human term syncytiotrophoblasts (hst), Chinese hamster ovary (CHO) cells, or LLC-PK porcine kidney cells is capable of conducting Na^+^, Ca^2+^, K^+^, Cs^+^, Ba^2+^, or Mg^2+^ ions [18,19,20]. The failure to conduct Ca^2+^ and disruptions in intracellular calcium signaling is associated with the etiology and pathology of ADPKD [21,22,23,24].

In addition to the variety of conductances of Pkd2/PC2, the protein has interactions with a large variety of partners that are implicated in the channel’s function. The most predominant partner is the polycystin-1 (PKD1) protein [9,10,11,12,25]. Other partners include TRP channel family members TRPC1 [26,27,28,29], TRPC4 [30], TRPV4 [27,31,32], as well as Pkd2 itself [29,33], and the inositol 1, 4, 5-triphosphate (IP3) receptor [34] or the ryanodine receptor (RyR3) [35] for intracellular calcium release. Data suggest that Pkd2 and the interacting partners form homo- or hetero- tetramers or homo-trimers [29,32,33,36,37,38]. PKD2 interacts with PC1 as a heterotetramer in a 3:1 PKD2:PC1 ratio [38,39,40]. To add to the complexity of the Pkd2 protein, the channel’s location and function are cell-type and membrane-specific [9,10,18,41,42,43,44,45,46]. 

In this study, we used a very versatile system to further investigate the location, function, and interacting partners of Pkd2 to provide new insights into this complex channel. The ciliated single-cell eukaryote *Paramecium tetraurelia* provides a formidable platform to study the Pkd2 channel in both the cilia and in the cell membrane. Its ciliary beating and behavior are driven by multiple ion channels, which is why its nick name is “swimming neuron”. *P. tetraurelia* provides a rich background to draw upon for Pkd2 function [47]. A single *Paramecium* cell is covered in a thousand or more cilia that can be easily separated from the cell bodies for investigation. The cells are amenable to RNA interference (RNAi) and over-expression of epitope-tagged proteins for localization studies using immunoprecipitations (IP) or immunofluorescence (IF). Electrophysiology and behavioral assays are used to investigate ion channel function, membrane permeability, and swimming speeds [48,49,50,51,52,53]. 

These studies were intended not only to elucidate function but also to uncover new interacting proteins by studying Pkd2 in a background that does not have homologous sequences for Pkd1 or the other TRP channels. We provide here the results of RNAi and epitope-tagged protein studies to investigate the Pkd2 channel in both the cell membrane and the cilia. We demonstrate that the *Paramecium* protein eccentric, XntA [54,55,56], is a potential interacting partner for Pkd2. Both Pkd2 and XntA are located at the cell membrane and in the cilia and both proteins appear to function in Mg^2+^-induced ciliary beating and swimming behavior. Depletion of *PKD2* or *XNTA* leads to less response to Mg^2+^ solutions and, likewise, less membrane permeability to Mg^2+^. Over-expression of the Pkd2 channel leads to more membrane permeability to Mg^2+^ and slower swimming speeds in Mg^2+^. We also demonstrate that over-expression of *PKD2*-FLAG can rescue the membrane permeability of ciliated XntA1 mutants to Mg^2+^. Based on the results, we propose that the Pkd2 channel is permeable to Mg^2+^ in the cell membrane and most likely in the cilia of *Paramecium*, demonstrating a novel role for this versatile channel.

## 2. Materials and Methods 

### 2.1. Cell Culture, Solutions, and Statistics

*Paramecium tetraurelia*, 51-s (sensitive to killer) or the *eccentric* mutant XntA1 were used for all studies [54,55,56]. Paramecia stocks and cultures were maintained at 15 °C, or 22–25 °C. Culture media [57] was inoculated with *Aerobacter aerogenes* and incubated at 37 °C for 24 to 48 h prior to use, unless otherwise noted (see RNAi by Feeding). All primers are listed in Table S1: Primers. 

Solutions for backward swimming assays, heavy metal toxicity, electrophysiology, and swimming speed contained a base buffer of 1 mM calcium citrate and ~1.3 mM Tris Base and prepared as previously described [53,58]. Additional salts were added before the pH was adjusted to 7.02 to 7.05 using 100 mM Tris Base: 25 mM TEA (tetraethylammonium chloride) with 5 mM MgCl_2_ (Mg/TEA); 4 mM KCl (resting buffer); 100 µM NiCl_2_; 5 mM KCl; 1 mM KCl; and 0.5 mM MgCl_2_ with 1 mM KCl. 

All studies were repeated a minimum of three times and statistical tests conducted are identified in the results and in the figure legends.

### 2.2. RNAi Construct Design and mRNA Depletion Using RNAi by Feeding

The sequence for *XNTA* (PTETG4300006001) is published [54] with no other closely related sequences within the *Paramecium* genome. Two paralog sequences exist for *PKD2* (*PKD2a*: GSPATG00005599001 and *PKD2b:* GSPATG00024265001) in the *Paramecium* Genome [59] and one more distantly related gene (*PKD2L*: GSPATG00012640001). *PKD2a* and *PKD2b* are over 84% identical at the nucleic acid level, and the construct used for RNA interference (RNAi) from GSPATG000055990001 targets both *PKD2a* and *PKD2b*. *PKD2L* is less similar, 47% identical at the nucleic acid level to *PKD2a* and *b*, and is not presented in this study. RNAi cultures were prepared as previously described [53,60].

Off-target analysis of the RNAi constructs using ParameciumDB [61] show the designed constructs only target the intended sequences with 1863 and 610 23-mer hits on *PKD2* and *XNTA*, respectively, with the exception of one 23-mer off-target by the *XNTA* construct. Representative images of depleted RNA levels for *PKD2* and *XNTA* depleted cells are shown in Figure S1: Reverse transcriptase PCR of *PKD2* and *XNTA* depleted cells. All control, *PKD2* depleted, and *XNTA* depleted cells were tested in Mg/TEA for backward swimming behavior before being used in experiments.

### 2.3. Swimming Behavior and Heavy Metal Resistance

Swimming behavior was tested as previously described [53] using the aforementioned solutions. Heavy metal resistance used NiCl_2_, based on previous studies, with the expectation that the XntA1 mutants would show a strong resistance to ciliary paralysis by this heavy metal [55]. Cells were briefly washed in Dryl’s solution (1 mM Na_2_HPO_4_, 1 mM NaH_2_PO_4_, 1.5 mM CaCl_2_, 2 mM Na-citrate, pH 6.8) followed by resting buffer. Ten cells were placed in each well of a 3-well depression slide containing NiCl_2_ in base buffer. Slides were maintained in a humidification chamber and the number of cells swimming in each depression was counted every 30 min to calculate the percentage of swimming cells. Each cell type was tested a minimum of four times. 

### 2.4. Creation of Epitope-Tagged Proteins, Microinjection, and Immunofluorescence

The generation of the epitope-tagged genes was done as described previously and cells were maintained as published [53,60]. The creation of the C-terminal 3×myc tag (3×EQKLISEEDL) using the pPXV plasmid (Courtesy of W John Haynes, University of Wisconsin, Madison, WI, USA) was done similarly to previously described methods [53] using the primers MYC TOP and MYC BOTTOM (Table S1: Primers) and the QuikChange Site-Directed Mutagenesis kit (Stratagene/Agilent, Santa Clara, CA, USA) as per the kits instructions using the Myc Mutate primer (Table S1: Primers).

Cells were collected and immunostained as published [53] using the following primary antibodies: rabbit anti-centrin, 1:1000 (*Tetrahymena* centrin, gift from Dr. Mark Winey, University of Colorado, Boulder, CO, USA) and mouse anti-Flag, M2 clone, 1:300 (Sigma, St. Louis, MO, USA). Cells were examined and images recorded using the DeltaVision Restoration Microscopy System (Applied Precision, LLC, Issaquah, WA, USA). All images were taken using a 60x oil-immersion objective and images were deconvolved and analyzed using SoftWoRx Pro software (Applied Precision). Images are stacks of 7 to 10 Z-sections to display immunostaining at the basal body, located just below the cell surface, and include some of the cilia just above the cell surface.

### 2.5. Cell Membrane and Cilia Isolations

For all preparations, control cells were expressing the epitope tag(s) and test cells were expressing the epitope-tagged genes. Cells were grown in four to six 1.5 L volumes of fortified wheatgrass medium at 22 °C–25 °C until cell densities reached approximately 8000–12,000 cells per mL. 

Cell membranes were collected, washed, and homogenized as done previously [62] and resuspended in IP500 buffer without detergents (500 mM NaCl, 100 mM Tris-Cl, 1 mM EDTA, 1 mM EGTA, 0.2 mM sodium vanadate, 10% sucrose, pH 7.5) [26]. Whole cilia were collected as previously described [63] with modifications. After collection, the cilia were resuspended in a small volume (100–200 µL) of membrane buffer (8 mM Tris, 50 mM KCl, 5 mM MgCl_2_, 1 mM EGTA, pH 7.4) without vortexing. For both cell membrane and cilia isolations, protein concentrations were determined using a BCA protein assay (Pierce/Thermo Scientific) followed by equalizing both test and control samples for volume and protein concentration using IP500 buffer. Five (cilia) or 10 µL (cell membrane) of each sample type was removed to be used as loading controls for western blot using Anti-Tubulin. Sufficient Triton X-100 and NP-40 were added to the isolated proteins to reach final concentrations of 1% and 0.5%, respectively. Fresh protease inhibitors (1 mM PMSF, 1 µg/mL Leupeptin, and 1 µg/mL Pepstatin) were also added to the samples before rocking on ice at 4 °C for 1 hr to solubilize. This was followed by centrifugation at 100,000× *g* at 4 °C in a Beckman L8-80 Ultracentrifuge for 45 min to remove insoluble proteins. The resulting supernatants were placed in sterile tubes for immunoprecipitation (IP). 

### 2.6. Immunoprecipitations (IP) and Western Blots

Using previously published methods, IPs used IP500 buffer containing detergents (1% Triton X-100 and 0.5% NP-40) in place of previously described buffers [53]. Where a second IP was performed using c-myc affinity agarose, the sample containing FLAG-affinity agarose was centrifuged at 250× *g* for 2 min after which the supernatant was moved to a fresh tube. Fresh protease inhibitors and 25 µL of prepared c-myc affinity agarose were added before rocking on ice at 4 °C overnight. The affinity agarose was then collected and washed as already described. Final IP agarose was mixed with 25–40 µL of 2× Sample buffer (approximate final concentration: 6.25 mM Tris Base, 1.5% SDS, 1% glycerol, 0.001% Bromophenol Blue, pH 6.8).

Samples were prepared and run on 7%–15% sodium dodecyl sulfate polyacrylamide gels (SDS-PAGE) as published previously [53]. Blots were developed using either Enhanced Chemiluminescence (ECL) (Perkin Elmer western Lightning® Plus-ECL) as per the kit instructions or alkaline phosphatase as described previously [64,65]. Primary antibodies: rabbit or mouse Anti-FLAG M2 clone (Sigma), 1:2500; rabbit or mouse Anti-C-myc (GenScript, Piscataway, NJ, USA), 1:2000; mouse Anti-Tubulin, acetylated (Sigma), 1:10,000.

### 2.7. Electrophysiology

Cells used for electrophysiology were in early logarithmic growth phase. Electrodes were pulled from glass with filament (World Precision Instruments, Sarasota, FL, USA) and back-filled with 500 mM KCl. Electrodes had a resistance between 40 and 110 MΩ. Resting membrane potentials were measured using a Warner Intracellular Electrometer IE-251A (Warner Instruments, Hamden, CT, USA) processed through AD Instruments PowerLab 4/35 and LabChart Pro (AD Instruments, Colorado Springs, CO, USA) as previously described [48,66]. The fluid in the recording chamber was under constant flow with a controlled rate of ~3 mL/min using a Buchler polystaltic pump (Buchler, Fort Lee, NJ, USA). Membrane potential (V_m_) was recorded continuously and V_m_ was noted at 4 min after each change of the solution, which is after the V_m_ had stabilized. The change in membrane potential (ΔV_m_) was calculated as the difference in V_m_ in 1 mM KCl versus 0.5 mM MgCl_2_ with 1 mM KCl.

Deciliated cells were impaled with the electrode within 10 min of being deciliated. Any cell that was able to move or produced an action potential during or after the recording (indicating cilia were present) were not included in the analysis. Cells were deciliated as published previously [67]. 

### 2.8. Swimming Traces

Swimming traces were done in a dark room using a Bausch and Lomb dissecting microscope fitted with a Canon EOS Rebel T6 digital camera. Cells were collected and incubated in 1 mM KCl in base buffer before being imaged. 30 µL of cells (containing ~25–40 cells) was placed on a large glass slide next to a 300 µL flattened pool of the test (0.5 mM MgCl_2_ with 1 mM KCl in base buffer) or control (1 mM KCl in base buffer) solution. Once the pools were joined, imaging began where the shutter was open for 2.5 sec and no more than seven photos were taken per trial. Traces were analyzed using ImageJ® [68] to convert pixels to mm/sec.

## 3. Result

### 3.1. Depletion of PKD2 Leads to Short Backward Swimming in Mg^2+^ and Resistance to Heavy Metal Paralysis

*Paramecium* swims by beating its many cilia strong toward the posterior of the cell. The speed of ciliary beating is dependent on the cell’s membrane potential (V_m_), which is governed by the cell’s permeability to ions in the environment. The V_m_ of *Paramecium* is primarily determined by the extracellular potassium ion concentration, allowing *Paramecium* to behave like a potassium electrode. These cells are also permeable to Na^+^ and Mg^2+^, but less so to Ca^2+^ and Cl^−^ ions [69,70,71,72].

Depolarization of *Paramecium* beyond threshold using high potassium, Mg^2+^, other permeable cations, or injected current opens the voltage-gated calcium channels (Ca_V_’s) of the cilia resulting in a graded action potential (AP). The AP allows Ca^2+^ to enter the cilia through the Ca_V_’s and reach the axoneme in sufficient amounts to change the ciliary power stroke. The change in ciliary beating causes transient backward swimming for as long as ciliary calcium remains high. The depolarization and AP are typically short, ending quickly through the activation of K^+^ channels in the cilia [73,74]. Because Pkd2 is a non-selective cation channel [9,18,20], we hypothesized that reduction of Pkd2 using RNA interference (RNAi) would reduce depolarizing conductances and alter the backward swimming behavior of depleted cells compared to control cells fed the empty RNAi vector. 

Our initial testing of *PKD2*-depleted cells suggested a Mg^2+^-specific phenotype, similar to the *Paramecium* mutant *eccentric* (XntA1, gift of Dr. R. Preston). The XntA1 mutant is impermeable to Mg^2+^, with no inward Mg^2+^ current and lacks the Ca^2+^-dependent Mg^2+^ conductance (I_Mg(Ca)_) [55,56,75]. The *XNTA* gene in the XntA1 mutant has a deletion creating a premature stop in the protein, severely truncating it at amino acid position 38. Expressing the wild type *XNTA* gene in the mutant returns I_Mg(Ca)_ and Mg^2+^-induced behavior, which led to XntA being called a magnesium-specific channel-like exchanger protein [54].

**Figure 1:**
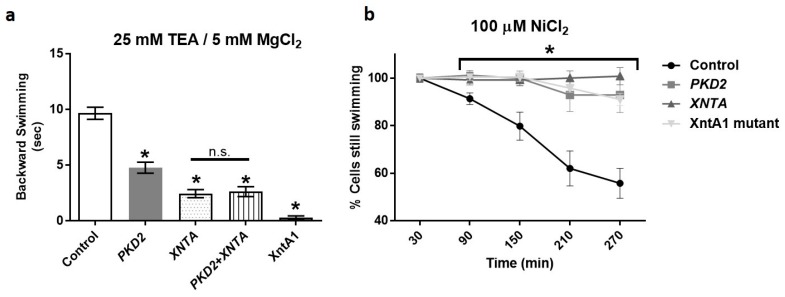
Depletion of *PKD2* shows a similar phenotype to *XNTA* depleted cells. (**a**) Average backward swimming times (sec) ± SEM of wild type (WT) cells fed the empty RNAi vector (Control), WT cells depleted of *PKD2*, *XNTA*, *PKD2* and *XNTA (PKD2*+*XNTA*), or XntA1 mutants in 25 mM TEA with 5 mM MgCl_2_. Cells depleted in *PKD2* show significantly shorter backward swimming compared to the controls (* = *p* < 0.01; T-test); cells depleted in *XNTA* or *PKD2+XNTA* show significantly shorter backward swimming compared to the controls or *PKD2* depleted cells (* = *p* < 0.01; T-tests) and are not significantly different from one another (n.s.). XntA1 mutants show significantly less backward swimming compared to all other cell types (* = at least *p* < 0.01; One-way ANOVA, multiple comparison tests). Experiments were repeated at least three times, N = 41–77 cells. (**b**) Heavy metal paralysis test of WT cells fed the empty RNAi vector (control), *PKD2* or *XNTA* depleted WT cells, or XntA1 mutants in 100 µM NiCl_2_. A minimum of 30 cells, 10 cells per well, were counted in each experiment (N = 12 to 21 wells, experiments repeated at least 4 times). Data are average percent of cells swimming ± SEM at different time points. Asterisk with bar indicates significantly different compared to control cells at and post 90 min (* = *p* < 0.001, ANOVA and multiple comparison tests).

Figure 1a shows that the backward swimming by the mutant XntA1 and wild type cells depleted of *XNTA* is much shorter than that of wild type control cells in 5 mM MgCl_2_ with 25 mM TEA (Mg/TEA) solution as we expect from previous work by Preston and Kung [55]. The XntA1 mutant shows almost no backward swimming in Mg/TEA which is significant compared to all other depleted cells (Figure 1a). Wild type cells depleted of *XNTA* using RNAi also show significantly shorter backward swimming in Mg/TEA compared to the control cells (Figure 1a).

Depletion of *PKD2* by the RNAi construct for *Paramecium PKD2* that targets both gene paralogs (*PKD2a* and *PKD2b*) also reduces backward swimming in Mg/TEA. The *PKD2* depleted cells showed significantly shorter backward swimming in Mg/TEA compared to control cells (Figure 1a) while concurrent depletion of *PKD2* and *XNTA* showed no additional decrease in backward swimming time compared to *XNTA* depleted cells (Figure 1a). 

A second XntA1 mutant phenotype we investigated was a resistance to heavy metal paralysis of the cilia. Exposure of *Paramecium* to heavy metals causes a decrease in ciliary beat and paralysis of the cells [76]. In mammalian cells, concentrations of 100 µM NiCl_2_ cause reduced metabolism and ATP content resulting in ciliostasis of rat, guinea pig, and hamster tracheal explants while producing no obvious changes in cell or ciliary morphology [77]. Previous studies of the XntA1 mutant demonstrated a strong resistance to ciliary heavy metal paralysis, especially in the presence of NiCl_2_, where XntA1 mutants were 10 times more resistant than the wild type cells to ciliary paralysis and death [55]. Therefore, we examined the resistance of wild type cells fed the empty RNAi vector (control), wild type cells depleted in *PKD2* or *XNTA*, and XntA1 mutant cells to heavy metal paralysis using 100 µM NiCl_2_. In Figure 1b, all data points for the XntA1 mutants and the *PKD2* or *XNTA* depleted cells at and post 90 min were significantly different compared to the control cells and the cells depleted of *PKD2* or *XNTA* or the XntA1 mutants were all equally resistant to 100 µM NiCl_2_ over time. 

### 3.2. Pkd2 and XntA Do Not Require Each Other for Trafficking

**Figure 2:**
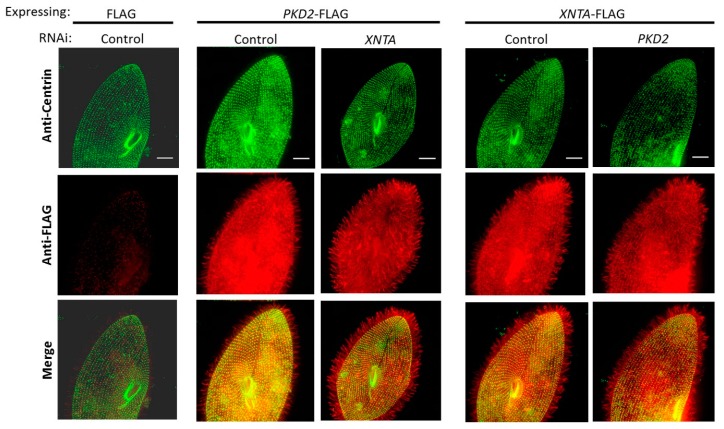
Pkd2-FLAG and XntA-FLAG are found in the cilia and at the cell surface while depletion of *XNTA* or *PKD2*, respectively, does not alter their location. Representative immunofluorescence images are stacks of 7 to 10 Z sections of cells over-expressing FLAG, *PKD2*-FLAG, or *XNTA*-FLAG fed RNAi bacteria before being collected and immunostained. Cells over-expressing FLAG fed the empty RNAi vector (control) serve as the negative control. *PKD2*-FLAG or *XNTA*-FLAG over-expressing cells fed the empty RNAi vector (control) serve as positive controls. Cells over-expressing *PKD2*-FLAG or *XNTA*-FLAG were depleted in *XNTA* or *PKD2*, respectively. Cells were stained with anti-centrin (green) to highlight the basal bodies just below the cell surface and with anti-FLAG (red) to show the epitope-tagged protein. Scale bars represent 15 µm.

Epitope tagging and immunofluorescence (IF) were used to localize the Pkd2 and XntA proteins in *Paramecium* and to determine if these two proteins require one another for trafficking. To determine whether Pkd2 requires XntA for localization or vice versa, we depleted *XNTA* or *PKD2* from cells expressing *PKD2*-FLAG or *XNTA*-FLAG, respectively, followed by IF to visualize changes in the location of the tagged protein. As a negative control, wild type cells expressing the 3×FLAG plasmid without an insert were fed the empty RNAi vector. As a positive control, cells expressing the epitope-tagged gene were fed the empty RNAi vector (control). The collected cells were stained with anti-centrin (green) (gift from Dr. Mark Winey, University of Colorado, Boulder, CO) to highlight the basal bodies below the cell surface and anti-FLAG (red) to locate the 3×FLAG epitope on the expressed protein. The results show the Pkd2-FLAG or the XntA-FLAG proteins at the cell surface and in the cilia (Figure 2). When *PKD2*-FLAG expressing cells were depleted of *XNTA*, or when *XNTA*-FLAG expressing cells were depleted of *PKD2*, there were no observable changes in the location of the Pkd2-FLAG or XntA-FLAG proteins, respectively. Likewise, no location changes of the epitope-tagged genes were observed when expressed in wild type cells or XntA1 mutants; the Pkd2-FLAG or XntA-FLAG proteins were seen in the cilia and at the cell surface (Figure S2: Pkd2-FLAG and XntA-FLAG are found in the cilia and at the cell surface and do not require each other for their localization). These results suggest the Pkd2 and XntA proteins traffic independently of one another and that these proteins are found in the same regions of the cell, the cilia and at or near the cell surface.

### 3.3. XntA-myc Co-Immunoprecipitates (co-IPs) Pkd2-FLAG 

**Figure 3:**
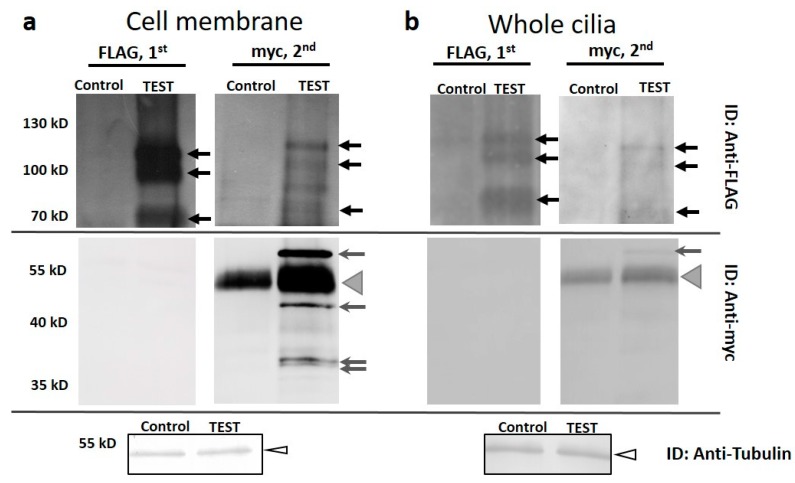
The XntA-myc protein can co-IP Pkd2-FLAG from solubilized cell membrane and whole cilia, however, the interaction occludes the FLAG epitope. IPs from isolated and solubilized (**a**) cell membrane or (**b**) whole cilia from cells expressing *PKD2*-FLAG and *XNTA*-myc (TEST) or the FLAG and myc epitopes (Control). First IP done using FLAG affinity agarose (FLAG, 1^st^) and after removal of the FLAG affinity agarose, the second IP from the same supernatant used myc affinity agarose (myc, 2^nd^). The resulting blots were used to probe for the Pkd2-FLAG protein (ID: Anti-FLAG; upper blots, black arrows) followed by stripping the blots and probing for the XntA-myc protein (ID: Anti-myc; lower blots, grey arrows). Note: the myc IP’s show the heavy chain of the antibody at 50 kD (grey arrow heads) and should be ignored. IPs targeting Pkd2-FLAG did not co-IP XntA-myc (ID: Anti-myc blots, FLAG, 1^st^ TEST lane). The IPs targeting XntA-myc (ID: Anti-FLAG blots, myc, 2^nd^ TEST lane) produced both XntA-myc (grey arrows, ID: Anti-myc) and Pkd2-FLAG (black arrows, ID: Anti-FLAG). Before solubilization, TEST and Control protein samples were equalized for protein amount and volume. Five µL was removed from both TEST and Control samples before solubilization and detected in a western blot using Anti-Tubulin (arrow heads, ID: Anti-Tubulin). Experiments were repeated three times for cilia and for cell membrane, representative blots are shown.

Because the Pkd2 and XntA proteins are both located in the cilia and at the cell surface and appear involved in the Mg^2+^ depolarization pathway, we investigated whether the Pkd2-FLAG and XntA-myc proteins interact to the extent that they might co-immunoprecipitate (co-IP). Cell membrane or whole cilia from wild type cells expressing *PKD2*-FLAG and *XNTA*-myc alongside control cells expressing FLAG and myc were isolated. The TEST and control samples were adjusted for equal protein concentration and volume before solubilization. A small sample was removed (5 µL) for western blot analysis using Anti-Tubulin to demonstrate the samples were of approximately equal concentration (see ID: Anti-Tubulin in Figure 3a,b).

Following solubilization, the insoluble proteins were removed by centrifugation and the resulting supernatant was IP’d from the solute first using FLAG affinity agarose. After the removal of the FLAG agarose, the supernatant was placed in a new tube and myc affinity agarose was added. Figure 3 shows the results of a representative FLAG IP (FLAG, 1^st^) followed by a myc IP (myc, 2^nd^) from solubilized cell membrane (Figure 3a) and whole cilia (Figure 3b). In Figure 3a, the Pkd2-FLAG protein is seen in the upper portion of the western blot (ID: Anti-FLAG). The Pkd2-FLAG protein appears as three bands at ~110 kD, 100 kD and 70 kD (ID: Anti-FLAG, black arrows). These three bands have been confirmed previously as Pkd2 using LC-MS/MS (data not shown). On the lower half of the same blot, no XntA-myc protein was detected (ID: Anti-myc). The second IP from the same supernatant targeting the XntA-myc protein (myc, 2^nd^) produced a band of the expected full-length XntA-myc protein (~63 kD) and smaller possible cleavage products at 45 kD and 37/38 kD (Figure 3a, ID: Anti-myc, grey arrows) in the lower half of the blot. The broad bands seen at ~50 kD (Figure 3a and 3b, grey arrow heads) in both the Control and TEST lanes is antibody heavy chain. (The antibody used for IP and ID were both produced in rabbit, and the heavy chain bands at 50 kD should be ignored.) The same IP sample where the XntA-myc protein was IP’d also produced bands for Pkd2-FLAG in the upper portion of the blot (ID: Anti-FLAG, black arrows). These data show we are able to co-IP Pkd2-FLAG when XntA-myc is the IP target, but when we IP Pkd2-FLAG, the XntA-myc protein does not co-IP. 

Similar results are seen for solubilized whole cilia from the dual expressing cells (Figure 3b). When we target the Pkd2-FLAG protein for IP, the Pkd2-FLAG protein (black arrows) is IP’d but there is no co-IP of XntA-myc. In the second IP when we target the XntA-myc protein (Figure 3b, myc, 2^nd^), we detect the expected full size XntA-myc protein at 63 kD in the lower half of the blot (ID: Anti-myc, grey arrow) and the Pkd2-FLAG protein in the upper half of the blot (ID: Anti-FLAG, black arrows). These data again demonstrate that we are able to co-IP Pkd2-FLAG with the IP of XntA-myc but not vice versa. In addition, to ensure the myc affinity agarose was not responsible for the IP of the Pkd2-FLAG, but that it was the XntA-myc protein, cells expressing only *PKD2*-FLAG were used in an IP first using myc affinity agarose followed by FLAG affinity agarose (Figure S3: Myc affinity agarose does not IP the Pkd2-FLAG protein (negative control)). The results demonstrate that the Pkd2-FLAG protein is IP’d not by the myc agarose, but most likely by the XntA protein. In the Discussion we propose that the Pkd2-FLAG protein interacts with XntA-myc at the C-terminus of Pkd2-FLAG, occluding the FLAG epitope in the interaction. This interaction makes it impossible for the anti-FLAG antibody to IP the Pkd2-FLAG protein.

### 3.4. Wild Type Cells Are Permeable to Mg^2+^ and XntA1 Mutants Are Not 

We next investigated the contribution of the Pkd2 and XntA1 proteins to Mg^2+^-induced behavior to tease apart the involvement of these proteins in Mg^2+^ permeability by using electrophysiology to measure membrane potential (V_m_) changes. We are not measuring current, but instead using the observed changes in membrane potential (ΔV_m_) between the resting V_m_ in 1 mM KCl followed by the newly established resting V_m_ in 0.5 mM MgCl_2_ with 1 mM KCl to infer membrane permeability. The difference between these two V_m_’s provides the calculated ΔV_m_ which we present as average ΔV_m_ in mV ± standard deviation (SD) except in figures where it is shown as ± standard error of the mean (SEM). While a positive ΔV_m_ is a depolarization and a negative ΔV_m_ is a hyperpolarization, only depolarizations were observed in this study. All ΔV_m_ averages as well as the average resting membrane potential of the cells in 5 mM KCl and 1 mM KCl are shown in Table S2: Average ΔV_m_ in 0.5 mM MgCl_2_ with 1 mM KCl and average resting membrane potentials of cells in 1 mM and 5 mM KCl.

**Figure 4:**
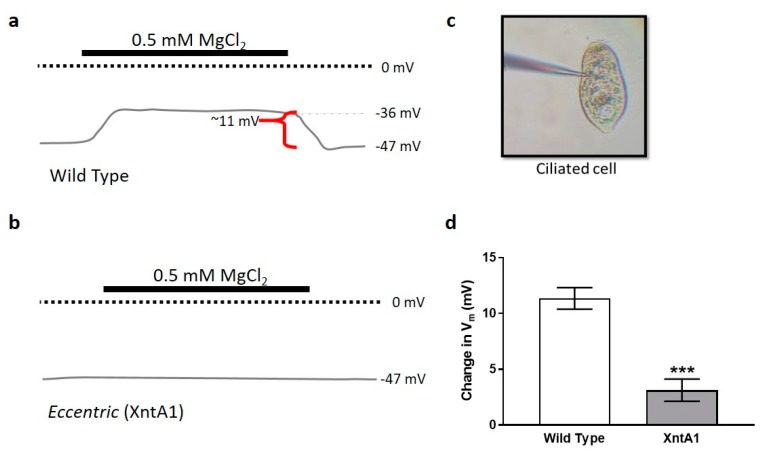
Membrane potential recordings show wild type cells are permeable to Mg^2+^ while XntA1 mutants are not. (**a**) Schematic representation of membrane potential recordings from wild type (WT) and (**b**) XntA1 mutants in 1 mM KCl, then 0.5 mM MgCl_2_ with 1 mM KCl, then returning to 1 mM KCl. Concentration of KCl (1 mM) remains consistent throughout the recordings, only the concentration of Mg^2+^ changes. Typical resting membrane potential of *Paramecium* (WT or XntA1) in 1 mM KCl is ~−47 mV. In the presence of 0.5 mM MgCl_2_, the membrane potential of WT cells depolarizes by ~11 mV while the XntA1 mutants show almost no change. (**c**) An XntA1 mutant during membrane potential recording. (**d**) As expected, wild type cells depolarize by 11.4 mV ± 2.7 (N = 9), significantly more than XntA1 mutants (3.1 mV ± 3.2, N = 11) in the presence of 0.5 mM MgCl_2_ (*** = *p* < 0.0001; T-test).

Previous work established that in the presence of 0.5 mM MgCl_2_ with 1 mM KCl, wild type cells depolarize by ~10 mV while XntA1 mutants, which are considered impermeable to Mg^2+^, depolarize by ~1.0 ± 7.0 mV (AVG ± SD) [56]. To remain consistent with these previously published findings, we used the same concentration of 0.5 mM MgCl_2_ with 1 mM KCl for our recordings. A schematic representation of a membrane potential recording from a wild type cell in 1 mM KCl followed by 0.5 mM MgCl_2_ with 1 mM KCl and returning to 1 mM KCl is shown in Figure 4a. By keeping the concentration of KCl constant, we are measuring the changes in V_m_ based on the presence or absence of MgCl_2_. The XntA1 mutant, however, is impermeable to Mg^2+^, and a recording of the membrane potential shows no change in the presence of MgCl_2_, as depicted in Figure 4b. A ciliated XntA1 mutant cell being recorded from is shown in Figure 4c. Our recordings recapitulate data published previously [56], showing similar changes in membrane potential of the wild type and XntA1 mutants (Figure 4d; Table S2: Average ΔV_m_ in 0.5 mM MgCl_2_ with 1 mM KCl and average resting membrane potentials of cells in 1 mM and 5 mM KCl). These data support our use of changes in membrane potential to infer membrane permeability to Mg^2+^.

### 3.5. The Amount of Pkd2 Is Important to Mg^2+^ Permeability

**Figure 5:**
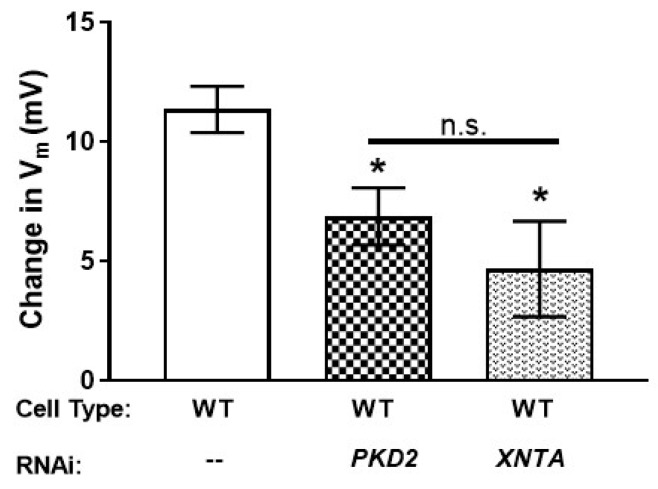
In wild type cells, depletion of *PKD2* or *XNTA* leads to less membrane permeability to Mg^2+^. Wild type (WT) cells were depleted of *PKD2* or *XNTA* (RNAi) and their membrane potential recorded in 1 mM KCl without, and then with, 0.5 mM MgCl_2_. Results are average ΔV_m_ in mV ± SEM (N = 7 to 9 cells). * = *p* < 0.05 compared to WT cells; n.s. with bar = not significantly different; T-tests.

We next depleted *PKD2* or *XNTA* from wild type (WT) cells, finding there was significantly less depolarization in the presence of Mg^2+^ compared to the control fed cells (Figure 5 and Table S2: Average ΔV_m_ in 0.5 mM MgCl_2_ with 1 mM KCl and average resting membrane potentials of cells in 1 mM and 5 mM KCl). These data agree with the backward swimming data where *PKD2* or *XNTA* depleted WT cells show short backward swimming in Mg/TEA (Figure 1a). In addition, depletion of *PKD2* from the XntA1 mutant produced no significant change in the amount of depolarization in the presence of Mg^2+^ (data not shown) compared to the XntA1 mutant (Figure 4d) or WT cells depleted in *XNTA* (Figure 5). Essentially, WT cells depleted in *XNTA* or *PKD2* or when XntA is absent (XntA1 mutants), cells depolarize significantly less than wild type cells in the presence of magnesium. 

**Figure 6:**
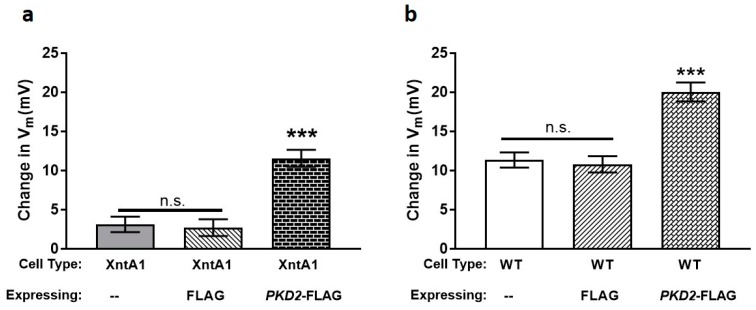
Over-expression of *PKD2*-FLAG restores Mg^2+^ permeability of XntA1 mutants to WT levels and increases membrane permeability to Mg^2+^ in WT cells. The average ΔV_m_ of (**a**) XntA1 mutant cells or (**b**) WT cells and those cell types over-expressing FLAG or *PKD2*-FLAG. Cells were recorded in 1 mM KCl without, and then with, 0.5 mM MgCl_2_. Results are average ΔV_m_ in mV ± SEM (N = 7 to 11 cells). *** = *p* < 0.0001 (T-tests) compared to (**a**) XntA1 mutants and XntA1 mutants over-expressing FLAG or (**b**) WT cells and WT cells over-expressing FLAG; n.s. with bar = not significantly different.

We then examined XntA1 mutants over-expressing FLAG or *PKD2*-FLAG to determine whether excess Pkd2 could impact the permeability of the cells to Mg^2+^ (Figure 6). XntA1 mutants and XntA1 mutants over-expressing FLAG were not significantly different from one another in their permeability to Mg^2+^, while the over-expression of *PKD2*-FLAG in XntA1 caused a significant increase in membrane permeability to Mg^2+^ (Figure 6a). This increase in depolarization by the XntA1 mutants expressing *PKD2*-FLAG (Figure 6a) is not significantly different compared to WT cells or WT cells expressing FLAG (Figure 6b; *p* = 0.888, T-test). These data demonstrate that over-expressing *PKD2*-FLAG in the XntA1 mutant results in a complete return of membrane permeability to Mg^2+^ to the previously impermeable mutant. In WT cells (Figure 6b), the over-expression of FLAG did not change the membrane permeability to Mg^2+^ compared the WT controls. The over-expression of *PKD2*-FLAG in WT cells dramatically increased membrane permeability to Mg^2+^, significantly more than WT cells alone or WT cells expressing FLAG (Figure 6b). These data suggest the amount of Pkd2 is critical to Mg^2+^ permeability of the cell.

### 3.6. Swimming Speed Is Slowed by Over-Expression of PKD2

**Figure 7:**
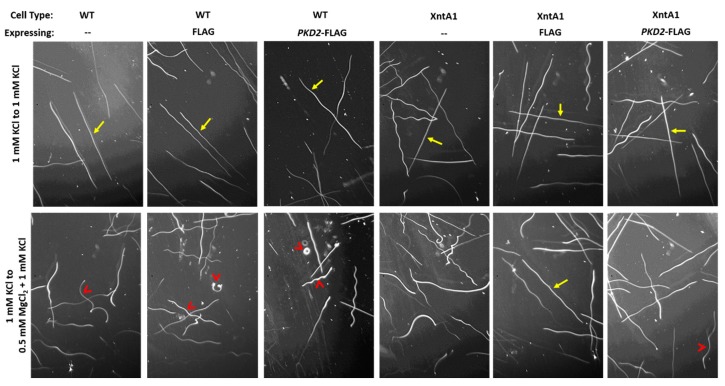
Representative swimming trace images of WT and XntA1 mutant cells and those cells over-expressing FLAG or *PKD2*-FLAG as the cells enter 1 mM KCl (control solution) or 0.5 mM MgCl_2_ with 1 mM KCl (test solution) from a 1 mM KCl solution. Cells were imaged in a dark field illuminated from the side using a 2.5 s exposure to appear white on a dark background. Traces were measured using ImageJ [68] and converted to mm/sec. Yellow arrows show straight, forward swimming traces, while red arrow heads show slower swimming traces with more turns.

To validate our findings of increased membrane permeability to Mg^2+^ by cells over-expressing *PKD2*-FLAG in both wild type and XntA1 mutants, we measured swimming speeds that reflect membrane potential and depolarization levels. Swimming speed is dependent upon the cilia whose beat frequency is governed by the membrane potential. Upon depolarization of the membrane, as in Mg^2+^ solutions, the cilia will beat more slowly causing slower swimming speeds [50,73,74,78]. The cells were collected and placed in 1 mM KCl before being imaged as they entered the test solution (0.5 mM MgCl_2_ with 1 mM KCl) or the control solution (1 mM KCl). Representative tracks of each cell type in the control and test solutions are shown in Figure 7.

**Figure 8:**
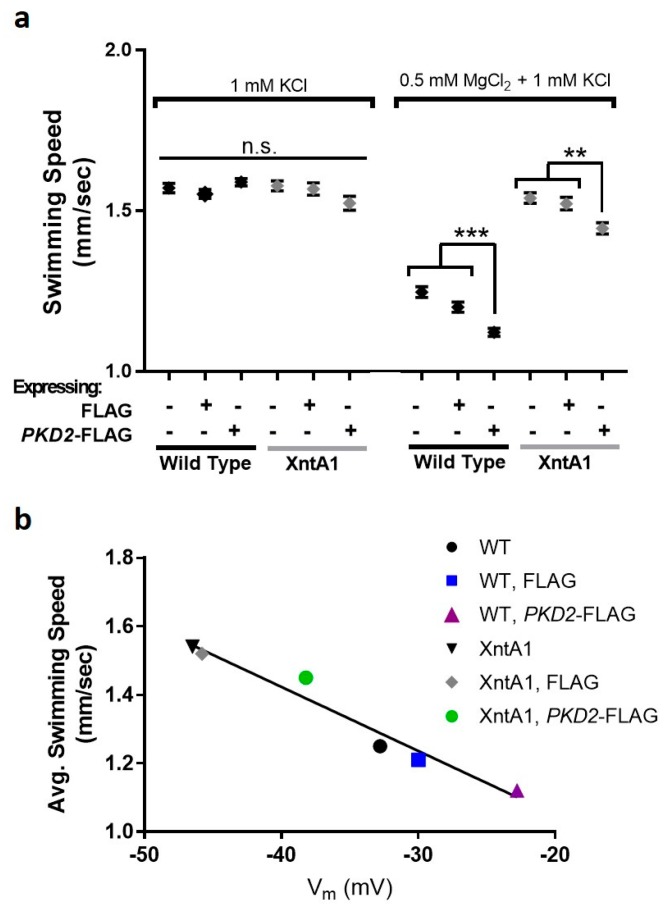
Over-expression of *PKD2*-FLAG significantly slows swimming speed in 0.5 mM MgCl_2_ with 1 mM KCl and average swimming speed is correlated with V_m_. (**a**) No significant difference (n.s.) is observed between wild type (WT), XntA1 mutants, or cells expressing FLAG or *PKD2*-FLAG when entering 1 mM KCl from 1 mM KCl. When cells enter 0.5 mM MgCl_2_ with 1 mM KCl from 1 mM KCl, WT and WT cells expressing FLAG significantly slow their swimming speed compared to entering 1 mM KCl. WT cells over-expressing *PKD2*-FLAG show significantly slower swimming than the WT or WT over-expressing FLAG in the Mg^2+^ solution (*** = *p* < 0.0001, T-test). XntA1 mutants and XntA1 mutants expressing FLAG do not change their swimming speed when entering 0.5 mM MgCl_2_ with 1 mM KCl from 1 mM KCl. Over-expression of *PKD2*-FLAG in the XntA1 mutants results in significantly slower swimming speeds compared to XntA1 and XntA1 expressing FLAG (** = *p* < 0.01, T-test). All data are average swimming speed in mm/sec ± SEM, N = 134–820 traces, each cell type was tested at least three separate times. (**b**) The swimming speed of the cells in 0.5 mM MgCl_2_ with 1 mM KCl is correlated with the membrane potential of cells in the same solution, linear regression R^2^ = 0.96.

When entering the control solution from 1 mM KCl, there was no significant difference between the swimming speeds of any of the cells, all cell types swam between 1.52 and 1.59 mm/sec (Figure 8a and Table S3: Average swimming speeds of cells from 1 mM KCl to either 1 mM KCl (control) or 0.5 mM MgCl_2_ with 1 mM KCl (test)). When WT cells or WT cells expressing FLAG entered the test solution, their swimming speed slowed significantly (Figure 8a). As expected, the XntA1 mutants and XntA1 mutants expressing FLAG did not slow their swimming speed when entering the test solution, as these cells are impermeable to Mg^2+^ (Figure 8a). 

The swimming speed of WT cells over-expressing *PKD2*-FLAG was significantly slower than that of WT cells or WT cells over-expressing FLAG (Figure 8a) validating the electrophysiology data where we claim a larger permeability to Mg^2+^ by cells over-expressing *PKD2*-FLAG (Figure 6b). Likewise, XntA1 mutants over-expressing *PKD2*-FLAG had significantly slower swimming in the test solution compared to XntA1 mutants and XntA1 mutants over-expressing FLAG (Figure 8a and Table S3: Average swimming speeds of cells from 1 mM KCl to either 1 mM KCl (control) or 0.5 mM MgCl_2_ with 1 mM KCl (test)). We show that the swimming speeds of the cells and their resting membrane potential in 0.5 mM MgCl_2_ with 1 mM KCl are well-correlated with a liner regression R^2^ = 0.96 (Figure 8b). The significantly slower swimming speeds of WT or XntA1 cells over-expressing *PKD2*-FLAG in the presence of Mg^2+^ (Figure 8a) combined with the electrophysiology data showing an increased membrane permeability to Mg^2+^ (Figure 6) strongly support the *Paramecium* Pkd2 protein as a possible Mg^2+^-permeable channel. 

### 3.7. Deciliated Cells Suggest Pkd2 Plays an Important Role in the Cell Body Membrane

We became interested in studying the activity of Pkd2 in the cell body membrane based on results from our previous publication where we examined the role of the BBSome coat complex in channel trafficking in *Paramecium*. In the study, we demonstrated that when we deplete *BBS8*, a component of the BBSome, Pkd2 is absent from the cilia. However, Pkd2 remains in the cell body membrane and the *BBS8* depleted cells display long backward swimming in Mg/TEA suggesting Pkd2 is active there [53]. To examine the role of Pkd2 in the cell body membrane without interference from the cilia, we applied our electrophysiology to freshly deciliated cells to infer membrane permeability to Mg^2+^. Paramecia are easily deciliated in 5 mM KCl with 5% ethanol and mild trituration which should not affect the resting membrane potential of the deciliated cells compared to ciliated cells [51]. We confirmed that all the deciliated cell types had the same resting membrane potential in 5 mM and 1 mM KCl as their ciliated cohorts (Table S2: Average ΔV_m_ in 0.5 mM MgCl_2_ with 1 mM KCl and average resting membrane potentials of cells in 1 mM and 5 mM KCl). 

**Figure 9:**
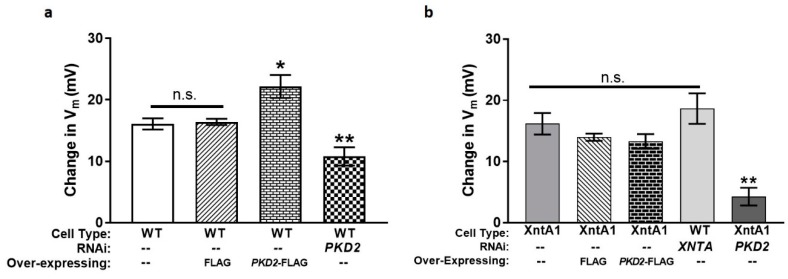
Depletion of *PKD2* in deciliated cells decreases the membrane permeability to Mg^2+^. Cells were freshly deciliated and recorded from in 1 mM KCl without, and then with, 0.5 mM MgCl_2_. (**a**) No significant difference was observed between deciliated WT cells and deciliated WT cells over-expressing FLAG (n.s. = not significantly different). Deciliated WT cells over-expressing *PKD2*-FLAG show a significant increase in Mg^2+^ permeability compared to deciliated WT and deciliated WT over-expressing FLAG (* = *p* < 0.05). Deciliated WT cells depleted in *PKD2* show a significant decrease in membrane permeability to Mg^2+^ compared to the others (** = *p* < 0.01). (**b**) Deciliated XntA1 mutants, deciliated over-expressing XntA1 mutants, and deciliated WT cells depleted in *XNTA* are not significantly different compared to one another (n.s.). Deciliated XntA1 mutants depleted in *PKD2* show almost a complete loss of membrane permeability to Mg^2+^ that is significantly different from all other deciliated cells types (** = *p* < 0.01). Data are average ΔV_m_ in mV ± SEM, N = 6–11 cells each, all statistics done using T-tests.

Surprisingly, deciliated WT cells showed a larger ΔV_m_ (Figure 9a) compared to ciliated WT cells (Figure 5; *p* < 0.01, T-test; and Table S2: Average ΔV_m_ in 0.5 mM MgCl_2_ with 1 mM KCl and average resting membrane potentials of cells in 1 mM and 5 mM KCl). While these data were unexpected, membrane potential recordings of deciliated cells in 0.5 mM MgCl_2_ with 1 mM KCl have not been done before, giving us no basis for comparison. Deciliated WT cells expressing FLAG were no different than deciliated WT cells (Figure 9a). Comparatively, deciliated WT cells over-expressing *PKD2*-FLAG show a significant increase in membrane permeability to Mg^2+^ (Figure 9a). Recordings of deciliated WT cells depleted in *PKD2* showed a significant decrease in membrane permeability to Mg^2+^ compared to deciliated WT control cells (Figure 9a; ** = *p* < 0.01, T-test). The increase and decrease in membrane permeability of deciliated cells over-expressing *PKD2*-FLAG and of deciliated *PKD2* depleted WT cells, respectively, suggests a critical role for the Pkd2 channel in Mg^2+^ permeability of the cell body membrane.

Upon recording from deciliated XntA1 mutants, we were surprised to find that these cells depolarized in Mg^2+^ by the same amount as deciliated WT cells (Figure 9a,b; *p* = 0.967, T-test). Deciliated XntA1 mutants expressing FLAG were not significantly different in their average ΔV_m_ compared to deciliated WT cells (Figure 9a,b; *p* = 0.07, T-test). Unexpectedly, deciliated XntA1 mutants over-expressing *PKD2*-FLAG showed no significant increase in membrane permeability to Mg^2+^ compared to deciliated XntA1 cells expressing FLAG (Figure 9b; *p* = 0.62, T-test). However, deciliated XntA1 mutants depleted of *PKD2* showed a dramatic loss of membrane permeability to Mg^2+^, significantly less permeability compared to the other deciliated cells presented here (Figure 9b; ** = *p* < 0.01, T-tests). Furthermore, note that the deciliated *PKD2* depleted XntA1 mutants are not significantly different in their permeability to Mg^2+^ compared to ciliated XntA1 mutants (Figure 6a; *p* = 0.536, T-test). These results suggest Pkd2 is necessary and sufficient for membrane permeability to Mg^2+^ of the cell membrane and that the XntA protein may play a regulatory role for PKd2 in the cell body membrane as well as in the cilia. 

## 4. Discussion

The depletion of *PKD2* in *Paramecium* produces a phenotype that resembles, but does not completely recapitulate, the phenotype of the XntA1 mutants. Comparison of *PKD2* depleted WT cells to both the knock-down of *XNTA* and XntA1 mutants demonstrated that *PKD2* depleted cells had a Mg^2+^-specific behavioral change and led us to focus on the role of Pkd2 as a potential Mg^2+^ channel. In vitro in other cell types, Pkd2 has been shown to be permeable to Mg^2+^ using isolated membranes enriched with ER from LLC-PK cells expressing human *PKD2*, but this permeability has never been demonstrated in vivo [20]. Because of the variety of behavioral changes demonstrated by the XntA1 mutant and *XNTA* depleted *Paramecium* cells, we question the previous classification of XntA as a Mg^2+^-specific channel-like exchanger [54]. Our data support that XntA is an important contributor to I_Mg(Ca)_, in agreement with previous data [54,56] and the data presented here demonstrate that ciliated XntA1 mutants are impermeable to Mg^2+^. However, our data implicate Pkd2 as a ciliary channel responsible for I_Mg(Ca)_, in combination with the XntA protein. 

The observed resistance to NiCl_2_ paralysis by *PKD2* or *XNTA* depleted cells suggests the loss of a major entryway for Ni^2+^, presumably through I_Mg(Ca)_. These results have been shown and proposed previously for XntA [55], but our finding that depletion of *PKD2* leads to a resistance of NiCl_2_ paralysis and our suggestion that Pkd2 is permeable to Ni^2+^ is novel. In mammalian cells, the melastatin TRP channels TRPM6 and M7 are highly permeable to Ni^2+^ and other heavy metals [79,80,81,82]. Also, TRPM6 and M7 form homo- and heteromeric complexes that are Mg^2+^-permeable, crucial for Mg^2+^ homeostasis in mammals [79,82,83,84,85]. There are no homologs for TRPM6 or M7 in *Paramecium*, however, based on the characteristics of TRPM6 and M7, we propose that Pkd2 in *Paramecium* is permeable to both Mg^2+^ and Ni^2+^.

In *Paramecium*, both Pkd2 and XntA appear to be in the same pathway and the presence or absence of XntA appears to have no impact on the trafficking or location of the Pkd2 protein and vice versa. In some mammalian cells, the trafficking of Pkd2 can require a signal from another protein, such as PKD1 [9,10]. However, Pkd2 and XntA do not appear to require each other or a signal from one another to traffic to the cell surface or to the cilia. It was not unexpected that Pkd2 in *Paramecium* would localize at the cell surface as well as in the cilia. It has been demonstrated that Pkd2 can localize to and be active in the cell membrane [9,18,41], the cilia [10,41,42] and the endoplasmic reticulum (ER) [20,43,44,86]. In some cell types, Pkd2 has been shown to require Pkd1 to be trafficked to another membrane as shown in Chinese hamster ovary (CHO) cells expressing human *PKD1* and *PKD2*. The Pkd1 protein recruits Pkd2 to the cell membrane where they form a functional channel [9]. In mouse embryonic kidney cells, changes in fluid stress cause Pkd1 to activate Pkd2 present in the ciliary membrane [10]. The data presented here is a similar scenario to that of *Caenorhabditis elegans,* where Pkd2 does not require Pkd1 to be trafficked to the cilia. In *C. elegans*, the homologue of Pkd1, *lov-1*, is not required for *pkd-2* to be trafficked to the membrane of ciliated neurons where these proteins are important for mating behavior [45]. Another instance is in the green algae *Chlamydomonas reinhardtii* where the Pkd2 protein is cleaved from a 210 kD protein into two smaller 120 and 90 kD proteins before entering the cilia completely independent of Pkd1 [46].

Our studies using the over-expression of epitope-tagged proteins combined with IP suggest the Pkd2 protein interacts with XntA, directly or indirectly, at the C-terminus of Pkd2, occluding the FLAG epitope. It is possible Pkd2 is interacting with other proteins at its C-terminus, however, we only examined XntA here. To summarize the findings shown in Figure 3a,b, and Figure S3, upon IP of the Pkd2-FLAG protein from solubilized cell membrane or whole cilia, there is no co-IP of XntA-myc. The second IP from the same solubilized sample targeting XntA-myc shows that XntA-myc is present and the co-IP of Pkd2-FLAG. Importantly, the IP of Pkd2-FLAG here was not due to the myc affinity agarose. Based on these results, we propose at least two different pools of the Pkd2 protein in the cell membrane or whole cilia. First, a fraction of Pkd2-FLAG exists with an exposed FLAG epitope, which explains our first IP where only the Pkd2-FLAG protein is visualized. Second, we propose the Pkd2-FLAG protein interacts with XntA-myc at the C-terminus of Pkd2-FLAG, occluding the FLAG epitope in the interaction. It is this second fraction of interacting Pkd2-FLAG that would permit the IP of XntA-myc and visualization of the Pkd2-FLAG protein. It is also this interaction that would prevent the co-IP of these two proteins using FLAG as the IP target. Another possibility is that the interaction between Pkd2-FLAG and XntA-myc is too weak to survive the IP process with FLAG as the IP target. However, we favor the prior explanation as the interaction, whether direct or indirect, is able to survive the myc IP process.

A C-terminal interaction for Pkd2 is unsurprising since mammalian Pkd2 is well-known for protein interactions through its C-terminal coiled coil domain, specifically with Pkd1 [1,8,9,12,40]. Our IP of Pkd2-FLAG from the same supernatant supports a second population of Pkd2 presumably from Pkd2 not interacting with other proteins, interacting at the N-terminus, or in a manner that leaves the FLAG epitope available for IP. Mammalian Pkd2 requires its N-terminus for the formation of homotetramers and for regulation [25,28,33]. While we did not examine other interactions here, we did express an N-terminal FLAG-tagged version of Pkd2 that caused short backward swimming in Mg/TEA compared to the FLAG expressing controls suggesting the N-terminal epitope tag interfered with protein function. The *Paramecium* Pkd2, like mammalian Pkd2, may require the N-terminus for homomultimer formation and proper channel function [33].

Our use of electrophysiology to measure ΔV_m_ in the presence of Mg^2+^ while keeping the K^+^ concentration constant allowed us to infer membrane permeability to Mg^2+^. In agreement with the backward swimming data, the depletion of *PKD2* or *XNTA* caused less Mg^2+^ permeability while over-expression of *PKD2*-FLAG led to increased Mg^2+^ permeability. This was especially true in the XntA1 mutant where the over-expression of *PKD2*-FLAG rescued the Mg^2+^-impermeable cells, returning Mg^2+^ permeability. We validated these findings by analyzing the swimming speeds of the over-expressing cells. We were gratified to see that the swimming speeds correlated with the recorded membrane potentials demonstrating that cells over-expressing *PKD2*-FLAG swim slower in Mg^2+^ and have larger membrane depolarizations compared to the FLAG-expressing control cells. Therefore, over-expression of *PKD2* increases membrane permeability to Mg^2+^, presumably due to an excess of Pkd2 channels. In addition, the over-expression of *PKD2*-FLAG in the eccentric mutant returns Mg^2+^ permeability suggesting that Pkd2, and not XntA, is sufficient for I_Mg(Ca)_.

Previously, we demonstrated that Pkd2 requires BBS8, a BBSome coat-complex protein, to reach the cilia. The *BBS8* depleted cells showed long backward swimming in Mg/TEA and Pkd2 sequestered at the cell surface [53]. These data suggest Pkd2 is functional at the cell surface, leading to our use of deciliated cells to examine Pkd2 activity in the cell membrane without interference by cilia or ciliary proteins. The observed increased permeability of deciliated cells to Mg^2+^, especially the deciliated XntA1 mutants, was unforeseen. With no functional XntA protein, our expectation was that, ciliated or not, the XntA1 mutants would not depolarize in the presence of Mg^2+^. Adding to the complexity of XntA, the absence of an increase in Mg^2+^-permeability by the deciliated XntA1 mutants over-expressing *PKD2*-FLAG was unexpected and unlike our observation of increased permeability by deciliated WT over-expressing cells. Possibly the XntA1 mutants were not sufficiently over-expressing *PKD2*-FLAG as we cannot control the level of over-expression. As an alternative explanation for the lack of increased permeability to Mg^2+^, we return to our suggestion that XntA has roles outside of I_Mg(Ca)_. The XntA protein may assist in stabilizing proteins, including Pkd2, in functional membrane microdomains. In deciliated XntA1 cells depleted of *PKD2*, we demonstrate almost a complete loss of Mg^2+^-permeability, suggesting Pkd2 is contributing to cell membrane Mg^2+^ permeability in the absence of XntA, but Pkd2 activity may be less steady. As demonstrated in mammalian cells, Pkd2 activity is stabilized though C-terminal interactions with Pkd1 [87] and Pkd2 interactions with α-actinin may help anchor the Pkd2 protein to the cytoskeleton to regulate signal transduction pathways [88]. Additionally, in human primary kidney epithelia cells, PC1 and PC2 (Pkd1 and Pkd2) are located in signaling microdomains marked by the protein flotillin-2 [89].

## 5. Conclusions

The outcomes of this study show a novel function of the Pkd2 protein in *Paramecium* as a Mg^2+^-permeable channel that is both necessary and sufficient for Mg^2+^ permeability and inferred I_Mg(Ca)_ function. The XntA1 mutant, which lacks I_Mg(Ca)_, can be rescued through the over-expression of *PKD2* or by deciliation. We have established the novel interaction of Pkd2 and XntA, either direct or indirect, in both the cell membrane and cilia. While this interaction is unnecessary for Pkd2 to function in the cell membrane, the presence of cilia or ciliary proteins stifles Pkd2 activity in the cell membrane. We propose a fresh responsibility for XntA outside of I_Mg(Ca)_, as a stabilizer for proteins, including Pkd2, in microdomains. Lastly, similar to mammalian cells, the amount of Pkd2 as well as its location are critical to the function of this versatile channel.

## Supplementary Materials

The following are available online at https://www.mdpi.com/2073-4425/10/6/455/s1, Figure S1: Reverse transcriptase PCR of *PKD2* and *XNTA* depleted cells; Figure S2: Pkd2-FLAG and XntA-FLAG are found in the cilia and at the cell surface and do not require each other for their localization; Figure S3: Myc affinity agarose does not IP the Pkd2-FLAG protein (negative control). Table S1: Primers; Table S2: Average ΔV_m_ in 0.5 mM MgCl_2_ with 1 mM KCl and average resting membrane potentials of cells in 1 mM and 5 mM KCl; Table S3: Average swimming speeds of cells from 1 mM KCl to either 1 mM KCl (control) or 0.5 mM MgCl_2_ with 1 mM KCl (test).
