# Supplementary material for: A Novel Role for Polycystin-2 (Pkd2) in P. tetraurelia as a Probable Mg2+ Channel Necessary for Mg2+-Induced Behavior
Source: Genes (Basel). 2019 Jun 14;10(6):455. doi: 10.3390/genes10060455 (PMC6627415; doi:10.3390/genes10060455)
Supplement: Supplementary file 1 [file genes-10-00455-s001.pdf]

genes

MDPI

Supplementary Material

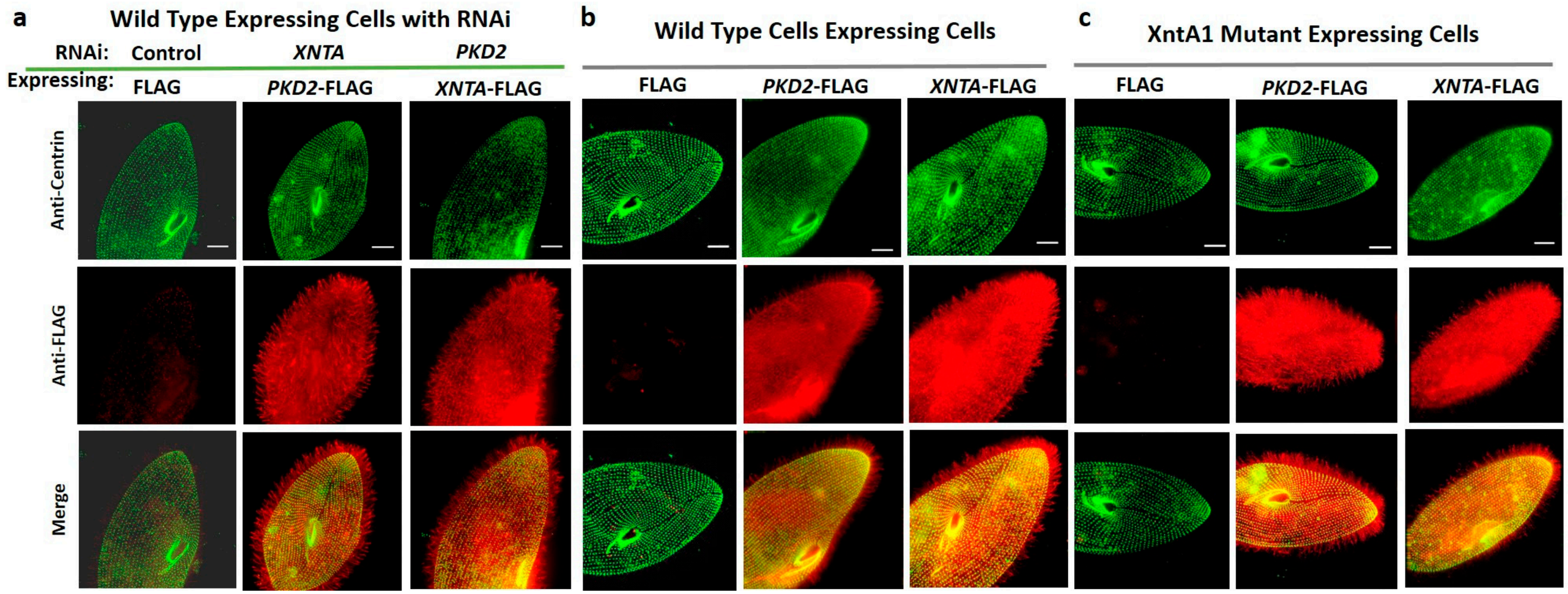

Figure S1. Pkd2-FLAG and XntA-FLAG are found in the cilia and the cell membrane and do not require each other for their localization. (a) Expression of FLAG (Control), *PKD2*-FLAG, or *XNTA*-FLAG in wild type (WT) cells fed RNAi for L4440 (Control), *XNTA,* or *PKD2*, respectively (from Figure 2). Cells were immunostained with anti-centrin to highlight the basal bodies just below the cell surface (green) and anti-FLAG to show the expressed protein (red). (b) WT cells expressing FLAG, *PKD2*-FLAG, or *XNTA*-FLAG to show the Pkd2-FLAG or XntA-FLAG proteins are located in the cilia and at the cell surface. (c) XntA1 mutant cells expressing FLAG, *PKD2*-FLAG, or *XNTA*-FLAG showing no change in the location of the expressed proteins in the mutant compared to WT cells. Pkd2-FLAG and XntA-FLAG are located in the cilia and at the cell surface. Cells are representative of the majority of the population imaged, scale bars represent 15 μm.

Table S1. Average ΔV_m in 0.5 mM $MgCl_2$ with 1 mM KCl and average resting membrane potentials of cells in 1 mM and 5 mM KCl.

**Ciliated cells**		**Resting Membrane Potential ± StDev (N)**	
**Cell Type**	**ΔVm ± StDev (N)**	**5 mM KCl**	**1 mM KCl**
Wild Type (WT)	11.4 ± 2.7 (9)	-27.7 ± 6.6 (20)	-46.4 ± 10.6 (20)
XntA1	3.1 ± 3.2 (11)	-30.7 ± 7.4 (17)	-49.4 ± 10.6 (16)
WT, *PKD2* RNAi	6.9 ± 3.4 (9)	-27.5 ± 8.9 (13)	-45.6 ± 10.3 (21)
WT, *XNTA* RNAi	4.7 ± 4.9 (7)	-26.3 ± 3.3 (9)	-44.3 ± 4.5 (13)
XntA1, *PKD2* RNAi	3.2 ± 1.7 (5)	-25.0 ± 3.0 (5)	-45.5 ± 6.5 (5)
**Expressing Cells**			
WT exp. FLAG	10.8 ± 2.6 (7)	-27.7 ± 6.6 (9)	-46.4 ± 6.2 (9)
WT exp. *PKD2*-FLAG	20.1 ± 3.2 (8)	-28.6 ± 6.9 (7)	-47.0 ± 6.3 (9)
XntA1 exp. FLAG	2.7 ± 3.0 (9)	-28.7 ± 4.0 (9)	-49.8 ± 6.9 (10)
XntA1 exp. *PKD2*-FLAG	11.6 ± 3.1 (9)	-29.7 ± 5.5 (7)	-53.1 ± 5.2 (8)
**Deciliated Cells**		**Resting Membrane Potential ± StDev (N)**	
**Cell Type**	**ΔVm ± StDev (N)**	**5 mM KCl**	**1 mM KCl**
Wild Type (WT)	16.1 ± 2.5 (9)	-28.4 ± 4.9 (16)	-47.5 ± 10.3 (11)
XntA1	16.2 ± 5.6 (11)	-30.6 ± 7.7 (16)	-49.5 ± 10.0 (19)
WT, *PKD2* RNAi	10.8 ± 4.2 (9)	-29.1 ± 6.0 (10)	-46.1 ± 8.6 (9)
WT, *XNTA* RNAi	18.7 ± 6.6 (8)	-27.6 ± 3.4 (13)	-48.9 ± 5.8 (15)
XntA1, *PKD2* RNAi	4.3 ± 3.3 (6)	-27.2 ± 4.4 (6)	-53.9 ± 5.2 (6)
**Expressing Cells**			
WT exp. FLAG	16.4 ± 1.4 (9)	-30.1 ± 6.1 (11)	-47.5 ± 6.8 (13)
WT exp. *PKD2*-FLAG	22.2 ± 5.3 (9)	-29.9 ± 6.5 (8)	-51.7 ± 7.8 (8)
XntA1 exp. FLAG	14.0 ± 1.7 (9)	-32.0 ± 4.9 (9)	-51.6 ± 7.1 (10)
XntA1 exp. *PKD2*-FLAG	13.3 ± 3.8 (11)	-32.0 ± 6.9 (12)	-53.4 ± 5.3 (13)

Table S2. Average Swimming speeds in mm/sec ± SD (N) as cells leave 1 mM KCl and enter either 1 mM KCl (control) or 0.5 mM $MgCl_2$ with 1 mM KCl (test).

**Cell Type**	**1 mM KCl to 1 mM KCl**	**1 mM KCl to 0.5 mM $MgCl_2$ with 1 mM KCl**
Wild Type (WT)	1.57 ± 0.18 (158)	1.25 ± 0.26 (227)†
WT exp. FLAG	1.55 ± 0.20 (224)	1.20 ± 0.31 (389)†
WT exp. *PKD2*-FLAG	1.59 ± 0.23 (406)	1.12 ± 0.36 (820)†, ***
XntA1	1.58 ± 0.22 (199)	1.54 ± 0.24 (213)
XntA1 exp. FLAG	1.57 ± 0.28 (225)	1.52 ± 0.31 (245)
XntA1 exp. *PKD2*-FLAG	1.52 ± 0.25 (134)	1.45 ± 0.25 (190)†, **

† = Indicates significantly different compared to the same cell type swimming speed in 1 mM KCl (***=P<0.01).

*** = WT expressing (exp.) *PKD2*-FLAG are significantly different compared to WT or WT exp. FLAG (P<0.001; T-tests).

**=XntA1 exp. *PKD2*-FLAG are significantly different compared to XntA1 mutants or XntA1 mutants exp. FLAG (P<0.01; T-tests).
